# The Late Queen-Empress: And after

**Published:** 1901-01-26

**Authors:** 


### The Late Queen-Empress: and After.

Our Queen-Empress has peacefully entered into rest, surrounded by her children and grandchildren—a fitting end truly to the Queen-Mother, whose sympathy with others and love for all her people will cause her name to be cherished and revered for ever. We have dealt in another place with the life and work of the Queen. Here we wish to try to realise the void which her death will cause, and to estimate how far her example and work are calculated to secure that that void shall be adequately filled, difficult as the task must necessarily prove. The influence which the Queen has exercised over us as a nation and in her family circle is the force best calculated to benefit her descendants and the English-speaking world. We can never forget, and the world can never forget, that the standard of virtue held up by the Queen has served to influence public opinion in the direction of all that is good and right, and public opinion is the lever whereby reform is gradually effected.

The more thoughtful have during the last few days been giving the closest consideration to the question of what is to follow the death of the Queen. At home the nation and the Empire look with confidence to His Majesty the King, and to his illustrious consort, to secure that the standard of virtue which has been the brightest feature of these realms under Victoria shall be continued and, if possible, extended year by year. If it be true—and it is true—that there is danger in over-preparation for more than half a lifetime, spent without direct and full responsibility, it is certainly to the honour of His Majesty the King-Emperor that his never-failing sense of duty and loyalty has sustained him for many years under circumstances where it was always difficult, and sometimes well-nigh impossible, not to make mistakes. For nearly sixty years His Majesty has lived amongst us, and has taken part in the life of the people, never sparing himself, or permitting anything on any occasion to interfere with the strict, adequate, and full discharge of the duties which have devolved upon him as Heir-Apparent to the throne. He has deservedly won the gratitude and affection of the people, and there is no family circle throughout the Empire which is more united or happy than His Majesty's. Only those who have an intimate knowledge of the inner life of the Royal Family can have any idea of the kindliness and affection which have won for him the hearts of his children and family, and the devoted adherence and loyalty of every member of his household. In matters of government and politics His Majesty has heretofore necessarily taken little or no public part, and the propriety of his attitude and the self-restraint which has invariably characterised his actions during so many momentous years are the soundest testimony to his high sense of duty and the capacity and intelligence with which he approaches affairs of State. Wherever it has been possible for him to use his influence to further the social well-being and comfort of the people, as in the matter of the better housing of the poor and the proper up-keep and adequate support of our hospitals, His Majesty has never failed to cheerfully give the whole weight of his influence to secure improvements and to promote progress. It would be difficult to over-estimate the importance of his public work, or of the example of personal service which His Majesty and Queen Alexandra have set to the nation, or of the value of the help they have rendered both to the social and charitable movements of our own times. It is but the truth to state that their Majesties have materially influenced for good the development and administration everywhere of the various institutions and social movements throughout the British Empire. We hope that they may both be long spared to continue that influence, and to fully and wisely develop every good enterprise of the kind for the benefit of the people.

At home His Majesty is well known and popular, and abroad his popularity is also deservedly great. We have reason to know that our King-Emperor is highly esteemed, not only by foreign peoples, but by their rulers, who in many cases are united to our own Royal Family by both affection and kinship. The action of the German Emperor in cancelling all the engagements of a highly important and pressing character which he had made in connection with the celebration of the bi-centenary of his family, and the despatch which he displayed in journeying to Osborne to see his grandmother the Queen before her death, will never be forgotten by the English nation; nor can the extent of the influence for good of this conduct be over-estimated. Our King-Emperor's action in going to Russia to support the present Czar through that trying fortnight after his father's death, in the discharge of what he considered a strict duty, was an example to men of all classes, which has already borne good fruit. We believe from our own observation in recent years in foreign countries that the mantle of our dear departed Queen-Mother will fall upon the shoulders of our King-Emperor, and that his accession and coronation will tend to strengthen the ties of sympathy which have for so long existed between other peoples and races, and make for the continuance of peace and the maintenance of principles which increase the happiness of nations.

And what of the Queen-Consort, who has from the time of her arrival on these shores been deservedly the most popular and best beloved of princesses? She is indeed a worthy successor to the Queen-Empress, as the embodiment of virtue, tenderness, and sympathy. The standard of virtue which she has well maintained and held up cannot fail to influence public opinion in the direction of all that is good and right, and so conduce to make the Court pure and entirely representative of all that is best, noblest, and most lovable. Every good work will have Her Majesty's staunchest support, and she has, too, what the Queen-Empress possessed to so high a degree, that love towards her Army and Navy, as well as towards all her people, which cannot fail to endear her to the nation, and make her a power for good in the land. Whilst we therefore mourn with heartfelt sorrow the lamented death of our much-beloved Queen-Mother, we can with full hearts and united voices in all sincerity say, "Long live the King-Emperor and his illustrious consort the Queen!"

